# A Web-Based Program for Cannabis Use and Psychotic Experiences in Young People (Keep It Real): Protocol for a Randomized Controlled Trial

**DOI:** 10.2196/15803

**Published:** 2020-07-29

**Authors:** Leanne Hides, Amanda Baker, Melissa Norberg, Jan Copeland, Catherine Quinn, Zoe Walter, Janni Leung, Stoyan R Stoyanov, David Kavanagh

**Affiliations:** 1 Lives Lived Well Group National Centre for Youth Substance Use Research, School of Psychology The University of Queensland Brisbane Australia; 2 School of Medicine and Public Health University of Newcastle Newcastle Australia; 3 Centre for Emotional Health Department of Psychology Macquarie University Sydney Australia; 4 Sunshine Coast Mind and Neuroscience - Thompson Institute University of the Sunshine Coast Birtinya Australia; 5 Centre for Youth Substance Abuse Research Centre for Children’s Health Research Queensland University of Technology Brisbane Australia; 6 School of Psychology & Counselling Queensland University of Technology Brisbane Australia

**Keywords:** cannabis, marijuana, substance use, psychotic, psychotic experiences, psychosis, digital intervention, web-based program, eHealth, adolescent, CBT, motivational interviewing, mindfulness, education, well-being

## Abstract

**Background:**

Young Australians (16-25 years) have the highest rates of past-month cannabis use in the world. Cannabis use increases the risk of alcohol and other drug disorders and depressive disorders, and has a robust dose-response association with psychotic experiences (PEs) and disorders. PEs are subthreshold positive psychotic symptoms, including delusions and hallucinations, which increase the risk of substance use, depressive or anxiety disorders, and psychotic disorders. Access to effective web-based early interventions targeting both cannabis use and PEs could reduce such risk in young people.

**Objective:**

The objective of this study is to determine the efficacy and cost-effectiveness of the *Keep it Real* web-based program compared to an information-only control website among young cannabis users (16-25 years) with PEs.

**Methods:**

Participants are recruited online, and consenting individuals meeting inclusion criteria (aged 16-25 years, who have used cannabis in the past month and experienced PEs in the past 3 months) are automatically randomized to either the *Keep it Real* web-based program (n=249) or an information-only control website (n=249). Both websites are self-guided (fully automated). The baseline and follow-up assessments at 3, 6, 9, and 12 months are self-completed online. Primary outcome measures are weekly cannabis use, PEs, and the relative cost-effectiveness for quality-adjusted life years. Secondary outcomes include other substance use and related problems, PE-related distress, cannabis intoxication experiences, severity of cannabis dependence, depression/anxiety symptoms, suicidality, and mental well-being and functioning.

**Results:**

Recruitment commenced in February 2019, and the results are expected to be submitted for publication in mid-2021.

**Conclusions:**

This study protocol describes a large randomized controlled trial of a new web-based program for young cannabis users experiencing PEs. If effective, the accessibility and scalability of *Keep it Real* could help reduce growing public health concerns about the significant social, economic, and health impacts of cannabis use.

**Trial Registration:**

Australian New Zealand Clinical Trials Registry ACTRN12618001107213; https://www.anzctr.org.au/Trial/Registration/TrialReview.aspx?id=374800

**International Registered Report Identifier (IRRID):**

DERR1-10.2196/15803

## Introduction

Young adults aged 18-24 years (23%) have the highest rates of past-year cannabis use in Australia [1]. This is alarming as cannabis influences cognitive and affective brain development [2,3], and accounts for 10.8% and 6.2% of the health burden in males and females, respectively [4]. It is estimated that one in six adolescents who try cannabis will develop dependence and daily users are at 18 times the risk [2,5,6]. Cannabis users are also at 3-4 times the risk of developing alcohol and other drug (AOD) use disorders, which increases to 8 times among daily users [2,5,6]. Any use and heavy use of cannabis also increase the risk of later depression symptoms and disorders (OR 1.17-1.62) [7]. Long-term use is associated with poor cognitive, educational, social, psychological, and physical health outcomes, which can have profound and lasting effects on the individual, his/her family, and the community [2,3,6].

Cannabis has a robust dose-response association with psychotic symptoms and disorders. A meta-analysis of 11 longitudinal population-based studies found that cannabis users were 40% more likely to report psychotic symptoms, and had *2.6 times* the risk of developing a psychotic disorder, with the level of risk increasing in a dose-response manner [8]. Heavy cannabis users have a fourfold risk of developing psychotic disorders [9]. Between 40% and 74% of young people with first-episode psychosis use cannabis, which has been associated with poorer clinical, social, and functional outcomes [10]. Cannabis use also increases the risk of psychotic relapse and hospital readmission, and cannabis cessation reduces the risk to the same level as nonusers [11-13]. Treatment-seeking samples of young people meeting ultrahigh-risk criteria for psychosis (ie, a family history of psychosis, brief intermittent psychotic symptoms, or attenuated psychotic symptoms) who heavily use cannabis and/or meet criteria for DSM-IV cannabis abuse or dependence are also more likely to develop a psychotic disorder (OR 1.75) [14,15].

Cannabis use has been strongly linked to psychotic experiences (PEs), which are subthreshold psychotic symptoms, including delusions and hallucinations. PEs are familial, heritable, and share many genetic, social, and environmental risk factors with the clinical phenotype of psychosis [16]. They are much more common in the general population than psychotic disorders (whose lifetime prevalence is 1%-3%), with an 11.9% median lifetime prevalence of self-reported PEs reported across 13 epidemiological general population cohort studies [17]. A 12-month prevalence rate of up to 28% have been reported in community samples of adolescents and young adults [18]. This is concerning, as a meta-analysis of six studies reported that people with PEs have 3.5 times the risk of developing later a psychotic disorder over a 3- to 24-year period, with the level of risk increasing with the severity/persistence of PEs [19]. PEs are also an important marker for other adverse mental health outcomes [16]. They have been found to triple the risk of later substance use disorders in a dose-response manner [20]. Young people with PEs are also at increased risk of current depression (6.5 times) or anxiety disorders (5 times), suicidal thoughts (3 times), and suicide attempts (4 times) [21-23].

Cannabis use has been strongly associated with PEs in general population samples. Up to 90% of past-week cannabis users reported PEs in the same week in an experience sampling method study [24]. A growing number of prospective studies have demonstrated a dose-response relationship between cannabis use and PEs in young people [25-27]. Changes in cannabis use have also been found to result in concomitant increases or decreases in PEs in a 5-year study among young adults [28]. A 20-year follow-up study found that weekly and occasional cannabis use in adolescence was associated with 4.3 and 2.3 times the relative risk of continuously high PEs, respectively [29]. This dose-response relationship between cannabis use and PEs has also been confirmed in biological and experimental studies using hair samples following smoking or intravenous administration of tetrahydrocannabinol (THC) [30].

Together, this research indicates that cannabis use is a preventable risk factor for PEs as well as for a range of other adverse substance use and mental health symptoms and disorders in young people [3]. A targeted early intervention that provides optimal treatment for cannabis use and PEs could reduce the risk of these adverse outcomes, and their associated personal, social, economic, and health costs [31]. However, less than 8% of people with cannabis use disorders [32], 13% of 18-year olds with PEs, and 52% of those with psychotic disorders have ever sought professional help [33]. Young cannabis users with PEs may be even less likely to seek help due to concerns about confidentiality and stigma. It is also unclear where young cannabis users with PEs should seek help. Mental health services may view PEs as substance related or not serious enough to warrant treatment. AOD services may also turn them away as their PEs may be considered too severe for AOD treatment settings. Web-based and mobile phone–based treatments provide an anonymous, and highly accessible way of delivering evidenced-based, high-quality psychological treatment to young people who are the most frequent users of the internet (United Kingdom: 100% males, 98% females; United States: 98%; Australia: 100%, 90% ≥3 times/day) [34-36].

Cognitive behavior therapy (CBT) has an established evidence base for multiple mental health difficulties. CBT effectively treats cannabis use when delivered face-to-face or via the web [37,38]. Web-based CBT programs also effectively reduce anxiety and depression symptoms and disorders [39]. Although systematic reviews have found web-/mobile-based programs for people with psychosis to be highly acceptable, usable, and engaging, and reduced the severity of psychotic symptoms and risk of transition to psychotic disorders among help-seeking patients at ultrahigh-risk for psychosis [40,41], no web-based early interventions for PEs have been tested in a randomized controlled trial (RCT) to date.

To address this gap, we developed the stand-alone *Keep it Real* (Version 1) web-based program which targets cannabis use and PEs in young people. The feasibility and outcomes of *Keep it Real* were tested in a pilot study among 213 young people (16-25 years), who had used cannabis in the past month and reported subthreshold PEs in the past 3 months (Community Assessment of Psychic Experience [CAPE-15] score of >18). The pilot study recruited 1089 past-month cannabis users over a period of 6 months using university student emails and social media and website posts. Almost all young people (95%) accessed the CAPE-15 PE feedback as well as a mean of 2.94 (SD 1.82) of the six *Keep it Real* modules. On average, they gave the program a rating of 4 out of 5 for overall objective quality on the eHealth Rating Scale (mean total score=3.82, SD 0.57), with good engagement (mean 3.29, SD 0.63) and high levels of functionality (mean 4.13, SD 0.72), aesthetics (mean 4.11, SD 0.68), and information (mean 4.14, SD 0.65) quality. High retention rates were achieved in the 3-month (n=200, 88.5%) and 6-month (n=180, 84.5%) follow-ups. Participants achieved significant reductions (all *P*<.001) in the frequency of cannabis use (Cohen *d* effect size=0.36, *d*=0.46) and related problems (*d* effect size=0.45, *d*=0.48) as well as the frequency of PEs (*d* effect size=0.67, *d*=0.70) and their associated distress (*d* effect size=0.33, *d*=0.40) at 3- and 6-months follow-up, respectively. Moderate effect sizes were found for reductions in cannabis use and related problems and PE-related distress, and large reductions in the frequency of PEs were found. Neither the frequency of cannabis use nor PEs at baseline had an impact on website engagement, retention rates, or treatment outcomes. Together, these results provide preliminary evidence that the *Keep it Real* web-based program could be effective and acceptable among young cannabis users with PEs.

This protocol paper describes an RCT aimed at determining the efficacy and cost-effectiveness of *Keep it Real* (Version 2) compared to an information control website (ICW) among young people: (i) aged 16-25 years, who have (ii) used cannabis in the past month, and (iii) experienced PEs in the past 3 months. It is hypothesized that *Keep it Real* will result in significantly greater improvements in primary outcome variables of cannabis use and PEs, and will be more cost-effective than the ICW control condition. Secondary outcomes such as cannabis, alcohol, tobacco, and other substance use and related problems, including PE-related distress, cannabis intoxication experiences, the severity of cannabis dependence, depression/anxiety symptoms, suicidality, mental well-being, and functioning; and baseline moderators of intervention effects (eg, childhood trauma, impulsivity, urbanicity, lifetime psychotic disorder) will also be examined.

## Methods

### Trial Design

**Figure 1:**
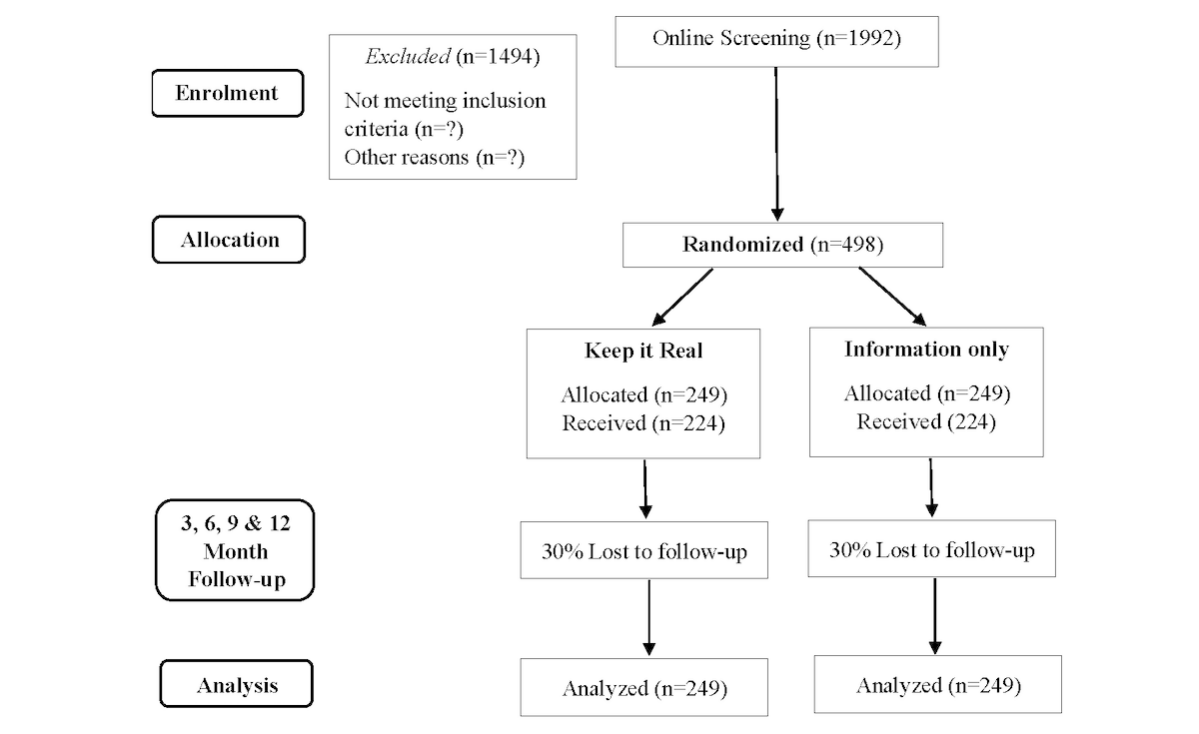
Anticipated CONSORT (Consolidated Standards of Reporting Trials) diagram.

The trial is a two-arm, parallel group (1:1 ratio) superiority RCT that will determine the efficacy and cost-effectiveness of the *Keep it Real* web-based program, compared to an ICW. Assessments are completed at baseline, and at 3, 6, 9, and 12 months follow-up. The trial has ethics committee approval, is registered (ACTRN12618001107213), and follows Standard Protocol Items: Recommendations for Interventional Trials (SPIRIT) research protocol guidelines (see supplementary materials) as well as Consolidated Standards of Reporting Trials (CONSORT) eHealth guidelines (see Figure 1) [42,43].

### Recruitment and Procedures

This project will utilize a secure, automated web-based project and data management system for RCTs. A two-step recruitment and informed consent process will be used. Young people aged 16-25 years who have used cannabis in the past month will be first asked to complete an online survey on *Cannabis and Psychotic Experiences*, and repeat the survey 6 and 12 months later. They are recruited online via university student emails and posts and paid advertising to social media (eg, Facebook) and substance use-related websites (eg, Cannabis Information Service) containing a link to an online consent form and survey. Snowballing techniques will be used and participants will be offered a reimbursement incentive of Aus $10 for referring a friend to the study. Consenting participants (via a checkbox) self-complete all baseline and follow-up assessments online. Survey logic automatically screens participants for eligibility. Participant contact details (name, email address, mobile number) and the IP address of respondents will be manually checked to identify repeat or duplicate surveys. Participants who give contact details that appear false, for example, multiple similar names or email addresses, will be excluded. All baseline, 6-, and 12-month survey completers will be entered in a draw to win 1 out of 10 Aus $100 gift vouchers.

Young people are asked if they are interested in trialing the *Keep it Real* website (via a checkbox) on the online survey consent form, and again at the end of the baseline survey. Those who express interest in trialing the website and score a minimum of 18 on the CAPE-15, which is indicative of at least three PEs “sometimes” or one PE “nearly always” in the past 3 months, are eligible to participate in the RCT. They are also required to have internet access and to provide a mobile number and email address. People who use alcohol or other illicit drugs are included as long as cannabis is the most frequently used drug (other than tobacco). Exclusion criteria will be (i) history of traumatic brain injury or organic brain disease, (ii) current acute suicide risk, and (iii) insufficient English fluency. Eligible participants are automatically sent the consent form for the RCT, and those who provide consent (via a checkbox) are randomized and sent an email and SMS link to either the *Keep it Real* web-based program or information-only control website. They are not informed about which website program they are allocated to. The study information sheet states that both programs provide information on PEs, psychosis, and cannabis use. They differ by their level of interactivity.

Young people who are ineligible for the RCT remain participants in the original survey study and are provided with immediate access to the *Keep it Real* website. Those in the RCT are automatically sent follow-up surveys at 3, 6, 9, and 12 months postbaseline via SMS and email. Noncompleters will be contacted via telephone by outcome assessors blind to treatment allocation to remind them to complete the survey online or over the phone. Participants are reimbursed Aus $20 for completing each follow-up survey (maximum of Aus $80). If a participant chooses to withdraw from the study, they will still be able to continue using *Keep it Real* or the ICW. The trial statistician will be blind to treatment group allocation.

### Measures

All measures are self-reported and the follow-up frequency is provided in Table 1. Demographic variables include sex, gender, occupation, education, employment, living arrangements, urbanicity, relationship status, migration status, ethnicity, estimated hours of internet use/day, family and personal history of mental and substance use disorders, and history of traumatic brain injury or organic brain disease.

**Table 1:** Assessment timeline.

Measure	Baseline	3 months	6 months	9 months	12 months
1. (Screen) Cannabis use in past month, age	✓				
2. (Screen) CAPE-15^a^	✓	✓	✓	✓	✓
3. Demographics	✓				
4. Psychosis screen	✓	✓	✓	✓	✓
5. Suicidality screen	✓	✓	✓	✓	✓
6. OTI^b^ for cannabis	✓	✓	✓	✓	✓
7. ASSIST^c^	✓	✓	✓	✓	✓
8. GAD-7^d^, PHQ-9^e^	✓	✓	✓	✓	✓
9. Cannabis knowledge	✓	✓	✓	✓	✓
10. SDS^f^	✓	✓	✓	✓	✓
11. SUPPS-P^g^	✓				✓
12. CEQ-I^h^	✓	✓	✓	✓	✓
13. MHC-SF^i^	✓	✓	✓	✓	✓
14. MAFS^j^	✓	✓	✓	✓	✓
15. CTQ^k^	✓				
16. EQ-5D-5L^l^ scale	✓	✓	✓	✓	✓
17. Health and service utilization	✓	✓	✓	✓	✓
18. eHRS^m^		✓			

^a^CAPE-15: Community Assessment of Psychic Experiences.

^b^OTI: Opiate Treatment Index.

^c^ASSIST: Alcohol, Smoking, and Substance Involvement Screening Test.

^d^GAD-7: Generalized Anxiety Disorder.

^e^PHQ-9: Patient Health Questionnaire.

^f^SDS: Severity of Dependence Scale.

^g^SUPPS-P: Short UPPS-P Impulsive Behavior Scale.

^h^CEQ-I: Cannabis Experiences Questionnaire-Intoxication.

^i^MHC-SF: Mental Health Continuum-Short Form.

^j^MAFS: Multidimensional Adolescent Functioning Scale.

^k^CTQ: Childhood Trauma Questionnaire.

^l^EQ-5D-5L: 5-dimension version of the European Quality of Life 5-level.

^m^eHRS: eHealth Rating Scale.

### Primary Outcomes

The primary outcome measures for cannabis use and psychotic like experiences are described below:

1. Cannabis use: The AOD section of the Opiate Treatment Index (OTI) will be used to assess the frequency (≤once a week; >once a week; ≥daily) and the mean amount of cannabis (in 1/4 gram standard units) per day in the past month [44]. As cannabis use is difficult to quantify, young people will be able to select their own cannabis unit (grams/cones/pipes/joints/$ spent/mixed with tobacco/shared), which will be converted into 0.25 gram cannabis units using standardized criteria [45].
2. Psychotic-like experiences: PEs are assessed with the 15-item version of the 20-item positive scale of the Community Assessment of Psychic Experiences (CAPE) measure. The CAPE-15 measures the frequency (1=never to 4=nearly always) and level of distress (1=not distressed to 4=very distressed) related to PLEs in the past 3 months [46]. This version has high levels of internal consistency and a more optimal three-factor structure, consisting of persecutory ideation, bizarre experiences, and perceptual abnormalities [46]. This 3-factor structure was replicated in a meta-analysis [47].

### Secondary Outcomes

A number of secondary outcomes will also be measured, as outlined below:

1. AOD-related problems: The 8-item Alcohol, Smoking, and Substance Involvement Screening Test (ASSIST) provides a measure of the frequency of substance use and related problems in the past 3 months for 10 substance classes [48]. It has well-established reliability and validity across a range of age groups and populations, including people with first-episode psychosis [48,49].
2. Depression/anxiety symptoms: The 9-item Patient Health Questionnaire (PHQ-9) and the 7-item Generalized Anxiety Disorder (GAD-7) scales will be used to assess depression and general anxiety symptoms in the previous 2 weeks. PHQ-9 and GAD-7 have been validated across a number of populations including adolescents, young adults, and substance users [50-53].
3. Suicidality: Suicidal ideation, plans, and attempts will be assessed using the 6-item Suicidality Scale of the Mini-International Neuropsychiatric Interview (MINI) [54].
4. Well-being: The 14-item version of the Mental Health Continuum-Short Form (MHC-SF) [55] ask participants about their emotional, psychological, and social well-being in the past month. The MHC-SF has been found to be reliable and valid in young adults [56].
5. Cannabis experiences: The Cannabis Experiences Questionnaire-Intoxication (CEQ-I) scale is a 13-item measure of the paranoid-dysphoric (PEs) and euphoric effects of cannabis intoxication validated in young adults [57].
6. Dependence: The Severity of Dependence Scale (SDS), a 5-item measure, will be used to assess the severity of psychological dependence on cannabis in the past 3 months. The SDS is validated for use in cannabis users and people with early psychosis [58,59].
7. Functioning: The Multidimensional Adolescent Functioning Scale (MAFS) is a 23-item self-reported measure of general, family, and peer-related functioning with good psychometric properties [60].

### Cost-Effectiveness

Health-related quality of life will be assessed using the 5-dimension version of the European Quality of Life 5-level (EQ-5D-5L) scale [61]. Information on other health care service use including medical and psychological treatment for substance use and other issues in the month prior to each assessment point will be collected.

### Other Measures

A number of other measures will also be included in the survey:

1. Lifetime psychotic disorders: The psychosis screen (patient form), which contains 6 items and up to 4 supplementary items for positive responses, will be used to identify lifetime psychotic disorders [62]. A cutoff score of ≥2, which has 67% sensitivity and 84% specificity, will be used [62]. Participants who answer positively to any of the items will also be asked to describe their experience in more detail in an open-ended response format. All responses will be checked prior to randomization to reduce the likelihood of false positives. A 3-month version of the psychosis screen will be used at follow-up.
2. Knowledge: In all, 19 questions will assess the young person’s level of knowledge about cannabis and its effects, with response options of “true” or “false.” The total score reflects the number of correct answers.
3. Impulsivity: The 20-item Short UPPS-P Impulsive Behavior Scale (SUPPS-P) provides a measure of the 5-factor model of impulsivity consisting of: (i) negative urgency (tendency to act rashly under extreme negative emotions), (ii) lack of premeditation (tendency to act without thinking), (iii) lack of perseverance (inability to remain focused on a task), (iv) sensation seeking (tendency to seek out novel and thrilling experiences), and (v) positive urgency (tendency to give into impulses when experiencing high positive affect) [63]. Higher levels of impulsivity and sensation seeking have been reported in first-episode psychosis in patients with rather than without cannabis abuse; and has been associated with PEs among young cannabis [64,65].
4. Childhood trauma: The Childhood Trauma Questionnaire (CTQ) is a valid and reliable 28-item measure of childhood abuse and neglect [66]. Childhood trauma will be included as a potential moderator, due to evidence that it may increase the risk of cannabis use, PEs, and other mental health issues [67].
5. Website evaluation: The 26-item eHealth Rating Scale, which is based on the Mobile App Rating Scale user version, will be utilized to measure the usability, engagement, aesthetics, information quality, and perceived impact of web-based programs at the 3-month follow-up [68]. Participants are also asked to provide a written feedback on the website.

### Randomization

A computerized random number sequence generator incorporating random permuted blocks will be used to allocate participants to: (i) *Keep it Real* or (ii) ICW. Stratification will be by cannabis usage (daily or less), sex, age (16-20 years and 21-25 years), and psychosis screen result (present vs not present). The randomization sequence is concealed within the secure website and the allocation is automatically released when full eligibility criteria are met.

### Interventions

Young cannabis users who meet RCT inclusion criteria and consent to participate are automatically randomized to either the *Keep it Real* or ICW program, and are sent an email and SMS link to the relevant program. Both programs are accessible via login on any computer, tablet, or mobile device with an internet connection. They are password protected and are sited on parallel, secure servers. Both programs are self-guided (fully automated), and all website modules are unlocked and can be accessed by the user at any time. SMS reminders to access both programs will be sent on day 7, 14, and 21 after baseline. Participants’ “backend” usage data will be automatically captured by the website, including logins, modules accessed/completed, inserted text, and dates of data entry. All participants have continuous access to the Get Help section of the website, which provides the contact details of mental health and AOD services (including crisis care).

#### Keep It Real Web-Based Program

The *Keep it Real* program (Version 2) consists of seven modules that can be completed in 30-90 minutes over 1-3 sessions (via login). Version 1 of *Keep it Real*, which was tested in the initial pilot study, only targeted cannabis use. Version 2 targets cannabis, alcohol, methamphetamine, and heroin use. Each module ends with a printable summary screen. PEs are targeted first, given young people are more likely to be concerned about PEs than their cannabis use. The first three modules aim to increase the users’ ability to identify, understand, and reduce distress associated with PEs. The program content was informed by the *Think You’re Crazy? Think Again* [69] self-help book, based on Morrison’s evidence-based CBT treatment for people at “ultrahigh risk” for psychosis (including those with PEs) [70-72]. Module 1 defines PEs and provides a detailed personal feedback on self-reported PEs relative to age-related and gender-specific norms. Module 2 provides information on risk factors for PEs (including cannabis use, trauma, stress, anxiety/depression), psychotic symptoms, and disorders using fact sheets and videos. Module 3 provides information and normative data on different subtypes of PEs and suggests a number of simple CBT techniques for their management. These modules were originally developed for the *Get Real* web-based program for young people with PEs. A small pilot study in 12 young people found that the program had high levels of acceptability and perceived utility, and resulted in significant reductions in the frequency and level of distress associated with PEs at the 3-month follow-up [73].

Modules 4 and 5 use motivational interviewing techniques to target cannabis, alcohol, methamphetamine, and heroin use. The program content was informed by evidence-based brief motivational interviewing interventions for cannabis use, including web-based programs [37,38,74]. A series of participatory design workshops with young people in residential treatment for substance use problems were also conducted to develop and refine the program content. Interactive quizzes on cannabis, alcohol, methamphetamine, and heroin use are included to increase their substance use knowledge. Young people are also given personal feedback on their substance use and related problems, relative to age-related and gender-specific norms. Participants can complete and receive normative feedback on the CEQ-I to increase awareness of the relationship between cannabis use and PEs [57]. They are then encouraged to set harm minimization or change goals and develop a plan for achieving them using harm minimization skill and change goal checklists.

Module 6 targets users’ coping skills by providing training in cognitive behavioral techniques including stress management, problem solving, behavioral activation, and attention control retraining (mindfulness). Module 7 encourages appropriate help seeking for PEs and/or cannabis use and addresses any barriers to doing so.

Participants can track their PEs (CAPE-15), cannabis intoxication effects (CEQ-I), substance use and related problems over time, and receive feedback on age-related and gender-specific norms each time they complete the measures. An interactive graphical summary of their PE, CEQ-I, cannabis, alcohol, methamphetamine, and heroin use and related problems over various time frames (self-selected) is also available to increase participants’ awareness of the relationship between their substance use and PEs.

#### Information Control Website

This program delivers the information sheets from the *Keep it Real* program in a 4-module web-based format including: (i) What are weird experiences? (ii) How does cannabis affect me? (iii) How does other substance use affect me? (iv) Should I seek help? Further reading sheets are also available from a “Fact Sheets” menu containing: (i) What is psychosis? (ii) Am I at risk of developing psychosis? (iii) What is schizophrenia? (iv) What is bipolar disorder? (v) Facts about cannabis/alcohol/amphetamines/heroin. This control condition was designed to provide participants with access to the type of web-based information they may have found naturally online. It does not provide personal feedback from assessments or normative feedback on substance use or PEs at baseline or follow-up. No information is given on how to manage PEs nor do the resources focus on building motivation to change substance use or increasing users’ understanding of the relationship between their substance use and PEs.

### Project and Risk Management

Weekly meetings monitor project implementation, clinical (including study withdrawals, adverse events), and research integrity (including data safety, important harms or unintended effects, blindness violations). Email or telephone consultations will be used to determine urgent issues, and a record of decision precedents will be kept to ensure consistency. Suicide and psychosis safety protocols identify and manage risk at all survey time points. Participants with a positive psychosis screen or who report a low level of suicide risk (MINI Suicidality Scale score of 1-5) receive a message in the online survey and an email providing details of appropriate support services and helplines. They are also asked to contact the research team if they have additional questions or would like assistance finding support. Those who report a moderate (Suicidality Scale score of 6-9) or high (10+) level of suicide risk are asked if they are receiving adequate support and if their feelings have recently improved (due to the 1-month time frame of the measure). If the participant answers yes to both questions, no further action is taken. If they answer yes to one of these questions or request further support, they are called within 48 hours by a research assistant to assess how further support can be provided (using a call script). Those who report a suicide plan or a suicide attempt within the past month are contacted by a trained clinical psychologist, who assesses the level of risk and helps the participant connect with appropriate supports.

### Sample Size Calculation

Power calculations to determine sample size were based on the effects found in the *Keep it Real* pilot study, which found a moderate effect size for reductions in the frequency of cannabis use (*d*=0.46). Due to the uncontrolled nature of the pilot study, a conservative approach using small to moderate effects was used. To measure a moderate effect size of *f*=.20, alpha (α) set at 0.025, and power set at 0.95, we would require 191 participants per group (a total of 382 participants). If baseline covariates accounting for about 40% of the variability in the outcome model were included in the calculations, then we would have 0.99 power to detect a small to moderate treatment effect. We predict a 20%-30% attrition rate at 12 months based on our pilot data and previous work and will therefore need to randomize a total of 498 participants.

The pilot study recruited 1089 past-month cannabis users over a period of 6 months (using the same recruitment strategies) to identify 860 monthly cannabis users meeting our PE inclusion criterion (CAPE-15≥18). Of these, 295 (34%) expressed interest in participating in the *Keep it Real* pilot trial (66 [7%] no interest; 503 [58%] no response) and 213 (72% of those interested; 25% of the total sample) were randomized. We will, therefore, need to recruit 1992 cannabis users in order to randomize 498 participants (see Figure 1).

### Data Analyses

The independent variable is a treatment condition with two levels: (i) *Keep it Real* and (ii) the ICW. The primary outcome variable is the frequency and average amount of cannabis/day (in 1/4 gram standard units) in the past month (on the OTI) and the frequency of PEs (CAPE-15, including subscales) in the past 3 months. Secondary outcome variables include the typical quantity and frequency of alcohol, tobacco, cannabis, and other drug use and related problems in the past 3 months (ASSIST), cannabis knowledge, PE-related distress (CAPE-15), cannabis intoxication experiences (CEQ-I), severity of cannabis dependence (SDS), depression (PHQ-9)/anxiety (GAD-7) symptoms, suicidality (MINI), mental well-being (MHC-SF), and functioning (MAFS). To determine whether there are group differences in the primary and secondary outcome measures at 3, 6, 9, and 12 months, a series of mixed effects models for repeated measures (MMRM) will be employed. The within-groups factor will be time (baseline, 3, 6, 9, 12 months) and group (*Keep it Real*, ICW) will be the between-subjects factor to examine the timexgroup interaction. This technique can also control for potential confounds (eg, other drug use and related problems) and examine potential baseline moderators of intervention effects (eg, age, gender, childhood trauma [CTQ], impulsivity [SUPPS-P], urbanicity, cannabis knowledge, lifetime psychotic disorder). Missing data will be handled using full information maximum likelihood estimation and an intention-to-treat analysis is performed.

A two-pronged economic analysis comparing *Keep it Real* and ICW will be conducted, taking a societal perspective, including costs of treatment provision and intervention (including website development and maintenance) as well as productivity costs. The analysis will display the cost-effectiveness in both natural units and as QALYs (based on the EQ-5D-5L). Models will assess *Keep it Real*’s offset on reduced health care service use and productivity losses are avoided. In a second modeled cost-effectiveness analysis (CEA), the estimated change in cannabis consumption from the trial will be extrapolated to the general Australian population of 16-25-year olds. Uncertainty will be assessed using a combination of nonparametric bootstrapping for the effect size (cannabis frequency) and Monte Carlo simulation for all other variables (with sampling uncertainty).

## Results

Recruitment commenced in February 2019, and is now closed for recruitment. The results are expected to be submitted for publication in mid-2021.

## Discussion

### Overview

Large numbers of young cannabis users report PEs, which increase their risk of developing psychotic, substance use, and depressive/anxiety disorders. Current services are failing to engage these young people—and would struggle to meet the need if they did seek help. An effective, safe, low-cost, nonstigmatizing accessible intervention is needed. Our pilot data showed that the *Keep it Real* program resulted in substantial reductions in both PEs and cannabis use, and young people found the program engaging and easy to use. This study protocol describes a large RCT that will determine if *Keep it Real* is more efficacious and cost-effective than the minimal web-based information they may otherwise receive.

### Strengths and Limitations

Engaging large numbers of young nonhelp-seeking cannabis users into web-based treatment is challenging, as they are unlikely to view their cannabis use as problematic or responsible for their PEs. However, the pilot study clearly demonstrated the feasibility of recruiting young cannabis users with PEs (79% of survey completers), with 72% of the 34% who expressed interest agreeing to be randomized to the trial. The pilot study also highlighted the acceptability of *Keep it Real*, as young people gave the program an average rating of 4 out of 5 for overall objective quality, and the functionality, aesthetics, and information subscales and a 3 out of 5 rating for engagement. Moreover, the pilot study provided preliminary evidence for the efficacy of *Keep it Real* as well as valuable data on the participation/retention rates and effect sizes to inform sample size which was used to ensure that this RCT is well powered.

Young people will be asked if they were interested in participating in the RCT prior to receiving feedback on their PEs. While this recruitment strategy will ensure that all young people are given the opportunity to participate, it may increase the risk of selection bias by potentially excluding those with little insight into their PEs. Nevertheless, the inclusion of people with a lifetime history of a psychotic disorder and the stratification of the randomization sequence by psychosis screen result may help reduce such risk of bias.

The inclusion of a range of potential control variables and moderators of treatment outcomes is a further strength of the study. However, mediators were not included due to concerns about potential assessment reactivity, the existing length of the assessment schedule, and participant burden. *Keep it Real* participants are able to monitor their PEs and substance use within the program. The extent of monitoring observed in this study will provide an indication of young cannabis users' willingness to engage in regular assessments for future studies exploring potential mediators of change.

The number of modules in the *Keep it Real* and ICW programs are not matched, but the ICW program contains the same number of information sheets as the *Keep it Real* program. While it could be argued that the smaller amount of content in the ICW may be insufficient to produce an effect, this control condition was designed to control access to a web-based program and the minimal amount and type of information users may otherwise receive online. Nevertheless, it is possible that the overlap in content may wash out any differential treatment effects between programs.

### Conclusion

Web-based programs are ideal platforms for delivering self-guided treatments to nonhelp-seeking young people with PEs. *Keep it Real* is the first web-based program to target PEs in cannabis users. The program content is based on evidence-based CBT treatments for people at ultrahigh risk for psychosis (which includes a subgroup with PEs) [41] and motivational interviewing interventions for cannabis use [37,38]. *Keep it Real* will be freely available and users will have unlimited 24/7 access to the program, including the PE and substance use monitoring and feedback, enabling the user to engage in continuous self-management. The program also has broad applicability as it can be used as a stand-alone treatment or as an adjunct to usual treatment for help-seeking cannabis users with PEs accessing clinical services. Different components of the program can also be used to address cannabis, alcohol, methamphetamine, or heroin use, PEs, cannabis intoxication effects, and to provide coping skills training. If *Keep it Real* demonstrates a substantial and cost-effective impact on cannabis use and PEs, its low unit cost and scalability would mean that it has substantial potential to make a population-wide impact on both immediate and longer term consequences of cannabis use and their significant social, economic, and health costs.

## Appendix

Multimedia Appendix 1 CONSORT-eHEALTH checklist (V 1.6.1).

## Abbreviations

AOD: alcohol and other drug
ASSIST: alcohol, smoking, and substance involvement screening test
CAPE: community assessment of psychic experiences
CBT: cognitive behavior therapy
CEQ-I: Cannabis Experiences Questionnaire-Intoxication
CTQ: Childhood Trauma Questionnaire
eHRS: eHealth Rating Scale
EQ-5D-5L: 5-dimension version of the European Quality of Life 5-level
GAD: Generalized Anxiety Disorder
ICW: information control website
MAFS: Multidimensional Adolescent Functioning Scale
MHC-SF: Mental Health Continuum-Short Form
MINI: Mini-International Neuropsychiatric Interview
OTI: Opiate Treatment Index
PE: psychotic experience
PHQ: Patient Health Questionnaire
RCT: randomized controlled trial
SDS: Severity of Dependence Scale
SUPPS-P: Short UPPS-P Impulsive Behavior Scale
